# Virtual Reality for Traumatic Brain Injury

**DOI:** 10.3389/fneur.2018.00345

**Published:** 2018-05-16

**Authors:** Elisa R. Zanier, Tommaso Zoerle, Daniele Di Lernia, Giuseppe Riva

**Affiliations:** ^1^Department of Neuroscience, IRCCS-Istituto di Ricerche Farmacologiche Mario Negri, Milan, Italy; ^2^Neuroscience ICU, Fondazione IRCCS Cà Granda – Ospedale Maggiore Policlinico, Milan, Italy; ^3^Dipartimento di Psicologia, Università Cattolica del Sacro Cuore, Milan, Italy; ^4^Applied Technology for Neuro-Psychology Laboratory, Istituto Auxologico Italiano, Milan, Italy; ^5^Centro Studi e Ricerche di Psicologia della Comunicazione, Università Cattolica del Sacro Cuore, Milan, Italy

**Keywords:** traumatic brain injury, virtual reality, brain protection, neurorepair, rehabilitation

## Abstract

In this perspective, we discuss the potential of virtual reality (VR) in the assessment and rehabilitation of traumatic brain injury, a silent epidemic of extremely high burden and no pharmacological therapy available. VR, endorsed by the mobile and gaming industries, is now available in more usable and cheaper tools allowing its therapeutic engagement both at the bedside and during the daily life at chronic stages after injury with terrific potential for a longitudinal disease modifying effect.

## Introduction

The World Health Organization estimates that traumatic brain injury (TBI) is and will remain the most important cause of neurodisability in the coming years (1). The search for neuroprotective therapies for severe TBI has been extensive but unfruitful over the last few decades, testified by more than 30 failed clinical trials, and we still have no specific neuroprotective therapy, that is, effective in clinical TBI. The burden of mortality and residual disability calls for new approaches to promote recovery of function of TBI patients in the acute and chronic phase (2, 3).

Classically described as a sudden event with short-term consequences, TBI induces dynamic pathological cascades that may persist for months or years after injury with a major impact on outcome (4, 5). Among dynamic mechanisms, the neuroinflammatory response and the accumulation of aberrant proteins may have a critical role in establishing a neuropathological link between acute mechanical injury and late neurodegeneration (6, 7). The close association between post-TBI neurological changes, persistent neuroinflammation, and late neuropathology highlights the fact that the window of opportunity for therapeutic intervention may be much wider than previously thought and that long-term treatment encompassing the acute and chronic phase should be tested to effectively interfere with this complex condition.

Importantly, next to the harmful processes, TBI also induces a neuro-restorative response that includes angiogenesis, neurogenesis, and brain plasticity (8, 9). These spontaneous regenerative mechanisms are short-lived and too weak to counteract damage progression but they could point the way to new therapeutic options if appropriately boosted and amplified. Physical and cognitive exercise increase repair and brain plasticity after injury in experimental models and patients (10, 11). Rehabilitative programs to provide inputs/stimuli to specific sensory or motor neural circuits, could in principle start very early on, and be finely tuned over time to account for the type and degree of injury and the level of motor and cognitive disability.

## Virtual Reality (VR) for Rehabilitation after TBI

Cognitive and physical rehabilitation programs are fundamental instruments to improve the clinical outcome of TBI patients optimizing the activities, function, performance, productivity, participation, and quality of life (12). They are based on restitutional, compensatory, and adaptive strategies and vary in relation to the patient potential and disability degree (2, 12).

Traumatic brain injury encompasses heterogeneous etiology, as well as structural and molecular patterns of injury dictating different prognostic features and potential responses to rehabilitative therapy. Experimental studies indicate that depending on the degree of cognitive and sensorimotor impairment exercise may improve outcome with different window of opportunity, however, evidence supporting the optimal timing, type, and intensity of rehabilitative interventions in patients are scarce (12, 13). For example, rehabilitation is often delayed in patients with severe TBI until their discharge from the intensive care unit, or adopted in the most severe cases with only minimal goals aimed at limiting spasticity (14). Importantly, cognitive rehabilitation in the sub-acute stage of TBI is rarely considered. For these reasons, the use of innovative techniques is advocated to assess the TBI-related deficits and to develop and evaluate new rehabilitative interventions (12).

An emerging technology, VR, represents a new tool for this purpose and might provide TBI care teams with new neuro-restorative strategies readily available at the bedside. Since the late 1980s, this term has been used to describe a 3D synthetic environment created by computer graphics, where the user has the feeling of being inside (15). VR can be described as “an advanced form of human-computer interface that allows the user to interact with and become immersed in a computer-generated environment in a naturalistic fashion” (16). For its flexibility, sense of presence (i.e., the feeling of “being there”) and emotional engagement, VR has been tested in motor and cognitive rehabilitation, with good results. In stroke patients, the number of VR programs is rapidly increasing with compelling data showing an improvement in recovery of motor function and daily living activities (17).

Data on the effects of cognitive function and quality of life are more limited. As underlined by two recent systematic reviews (18, 19), VR allows a level of engagement and cognitive involvement, higher than the one provided by memory and imagination, but is more controlled and can be more easily measured than that offered by direct “real” experience. Its multisensory stimulation means VR can be considered an enriched environment that can offer functional and ecological real-world demands (e.g., finding objects, assembling things, and buying stuff) that may improve brain plasticity and regenerative processes (20–22).

There are several examples in the literature where VR has been successfully used both as assessment instrument and as therapeutic intervention. As assessment tool, VR has been used to detect visual-vestibular deficits in adults after concussion and mild TBI (23, 24). Wright WG et al., developed a Virtual Environment TBI Screen that allows subjects to explore a digitalized setting (i.e., outdoor Greek temple with columns, different kind of floor materials, etc.) performing postural tasks while the system collects data to detect visual-vestibular deficits. Besnard et al. (25) created a virtual kitchen to assess daily-life activity and evaluate executive dysfunctions in subjects with severe TBI. Robitaille et al. (26) developed a VR avatar interaction platform to assess residual executive functions in subjects with mild TBI. The platform can capture real-time subject’s movements translating them in to a virtual body, that is, therefore placed in a simulated environment (i.e., a village). The user is then allowed to explore the simulate surroundings which comprise different navigational obstacles to overcome. Similar approaches have been used by other authors, whereas simplified settings (i.e., 3D virtual corridor that the subject can explored with a joystick) have been proved useful to assess subclinical cognitive abnormalities in asymptomatic subjects that suffered a concussion (27).

As therapeutic instrument, Dahdah et al. (28) demonstrated that immersive VR intervention can be used as an effective neuro-rehabilitative tool to enhance executive functions and information processing in the sub-acute period, providing evidence of positive effects of a virtual Stroop task over traditional non-VR-based protocol. VR as therapeutic instrument has also been used for attention training in severe TBI with positive results in the early recovery stages (29) with a specific “augmented” task in which virtual and haptic feedbacks were used in a target-reaching exercise to enhance sustained attention. Finally, virtual protocols generated upon commercial available game solutions have been effective in addressing and treating balance deficits (30).

All these works suggest that VR could be useful as assessment instrument and in the rehabilitation of TBI, nonetheless a delineated pattern seems to emerge. VR assessment protocols appear to be primarily implemented for mild TBI, which induce subtle residual deficits hard to detect with traditional instruments (23). Conversely, VR treatment protocols for cognitive rehabilitation are used transversely from mild to severe conditions, although effectiveness of these kinds of interventions needs to be further explored (31).

## Limitations and Future Directions

The use of VR in clinical practice has been limited by two main factors: accessibility and the cost of virtual tools. Nevertheless, VR technology is advancing quickly. Oculus Rift© and HTC Vive™ have showcased high-quality VR experiences at reasonable prices—less than $3,000 for a fully configured system—that should be widely available to consumers within this year (32), and even more affordable solutions based on smartphones and tablets are on the way (see Table 1). New interaction paradigms, like eye tracking, are allowing the use of VR also at the bedside in patients with limited mobility (32). The potential for activity-dependent structural and functional brain remodeling in behaviorally unresponsive brain-injured patients for up to 5 years has recently been shown (33).

**Table 1:** The most commonly virtual reality (VR) systems.

	VR systems									
	
	CAVE		PC based			Mobile based		Console based	Standalone	
User mobility required	Low/medium/high	Low/medium	Low/medium/high	Low/medium	Low	Low	Low	Low	Low	Low/medium

System name (cost)	Proprietary (>100,000 $)	Oculus Rift (500–1,000 $)	HTC Vive (500–1,000 $)	Microsoft (200–500 $)	Samsung Gear VR (<200 $)	Google Cardboard (<100 $)	Google Daydream (<200 $)	Playstation VR (200–500 $)	Oculus Go (<200 $)	Mirage Solo (400–500 $)

Hardware requirements (cost)	High end PCs (>1,000 $)	High end PC (>1,000 $)	High end PC (>1,000 $)	Mid level PC (500–1,000$)	High end Samsung phone (500–1,000 $)	Middle/high end Android phone or iPhone (<500 $)	High end Android phone (<500 $)	PS4 or PS4 Pro (<500 $)	None	None

Body tracking	High	Medium	High	Medium	Low	Low	Low	Medium	Low	Medium

User interaction with VR	High	High	High	High	Medium	Low	Medium	High	Medium	Medium

Software availability	Custom build applications	Oculus store	Steam store	Windows store	Oculus store	Google Play or IOS store	Google Play	Playstation store	Oculus store	Google Play

*The costs are based on information provided by system vendors in most European and North-American countries and they are expressed in USA dollars. *User mobility required*: low: static position, medium: limited movements in the space, high: active movements in the space. *Body tracking*: low: head tracking (rotation), medium: head tracking and positional tracking (forward and backward), high: head tracking and volumetric tracking (up to full room size movements). *User interaction with VR: high*: using joystick and/or controllers, medium: using gaze, a built in pad or joystick, low: using gaze or a bottom*.

*CAVE, cave automatic virtual environment; PC, personal computer*.

Literature evidence suggested that VR protocols can provide innovative assessment and treatment options for TBI, nonetheless possible limitations connected to perception of VR technology and usability, especially in older adults must be taken into account. TBI has a second peak of incidence in the elderly (2). This introduces a challenge related to the limited experience that elderly subjects have with new technological devices. However, evidence indicated the feasibility of VR interventions in elderly across different pathologies (34) even with active compromised spatial abilities and degenerative cognitive diseases (35), whereas different learning curves due to age-related differences have been effectively addressed through a training phase assisted by an expert (34). Finally, common side effect of VR intervention (i.e., motion sickness and disorientation) did not appeared to be specifically related to age (36), thus supporting the feasibility of VR protocols in aged patients.

Time is a key issue in TBI, with a window of vulnerability and opportunity that appears much wider than previously thought: this provides an incentive to look for continuous long-lasting therapeutic interventions to interfere with neurodegenerative processes and promote regeneration. From this viewpoint, VR offers a new strategy to boost and amplify restorative processes in the clinical setting at early stages of the disease, and in daily life at later stages (26). As discussed, VR allows the development of real-life, context-specific experiences, requiring the control of the individual over different cognitive sensorimotor, and social factors, which are usually difficult to reproduce in a clinical setting. For example, VR is effective in assessing a patient’s ability to perform everyday activities like cooking in a virtual kitchen, driving a virtual car, or shopping in a virtual supermarket. In these challenging but ecologically valid VR environments, behaviors can be assessed and trained while maintaining experimental control over stimulus measurement and delivery.

In general, the greatest long-term burden to patients are deficits in cognition and behavior (5). Here too, later VR interventions, with a focus on memory, attention, executive function, behavioral control, and regulation of mood, may be helpful in reducing the long-term problems and disabilities experienced by subjects after a TBI.

## Conclusion

In conclusion, VR has the potential for improving the assessment and treatment of TBI even in cases where the chances of recovery appear poor. The mobile and gaming industries are now significantly endorsing this technology, producing more and more usable and cheaper tools, that can be employed even at the bedside. Thus, collaboration between clinicians, researchers, and technology developers is required to produce VR tools that can fully exploit the terrific potential of this technology in TBI patient.

## Author Contributions

EZ, TZ, DL, and GR contributed to the conception and writing of the manuscript. All authors read and approved the final manuscript.

## Conflict of Interest Statement

The authors declare that the research was conducted in the absence of any commercial or financial relationships that could be construed as a potential conflict of interest.
